# Assessment of medical students’ attitudes on social media use in medicine: a cross-sectional study

**DOI:** 10.1186/s12909-015-0300-y

**Published:** 2015-02-15

**Authors:** Kadriye Avcı, Sevda Gerek Çelikden, Semih Eren, Doğukan Aydenizöz

**Affiliations:** 1 Department of Public Health, Faculty of Medicine, Afyon Kocatepe University, Afyonkarahisar, Turkey; 2 Afyon Kocatepe University, Faculty of Medicine, 5th Grade Medical Student, Afyonkarahisar, Turkey

**Keywords:** Social media, Medical education, Ethics

## Abstract

**Background:**

Social media has created a revolution in health services. Information available on the Internet and via social media is now being used as reference guides for sensitive health issues by nonprofessionals, physicians, and medical students. When used by physicians and medical students, social media has the potential to raise issues such as the blurring of the line between professional and private lives, patient relations, and medical ethics. The aim of this cross-sectional study was to evaluate the use of social media and attitudes toward its use in medicine among medical students.

**Methods:**

Medical students from Afyon Kocatepe University, Faculty of Medicine (Afyonkarahisar, Turkey) were asked to participate in a survey consisting of two sections, the first containing questions assessing the frequency of social media use and the second regarding attitudes toward the use of social media in medicine.

**Results:**

Survey responses indicated that 93.4% of medical students used social media and 89.3% used social media for professional purposes. Factor analysis showed that attitudes toward social media are based on five factors: professional usefulness, popularity, ethics, barriers, and innovativeness. A structural equation model revealed the highest positive correlation between usefulness and innovativeness; ethics had a low but positive correlation with other factors.

**Conclusions:**

Although social media is being used extensively by medical students, they appear unaware of possible ethical issues. Therefore, social media guidelines should be developed.

## Background

Although the advent of new media technologies such as the Internet and social media provide exciting opportunities to facilitate and enhance worldwide communication, uncertainty also remains regarding potential negative consequences. Because the number of social media users and the scope of their use has increased, significant research attention is now being paid to the use of social media in daily life and the complex interaction between its use and behavior in other domains. The distinction between public and private life are changing as a result of social media, causing some to question the merit of this form of communication [1]. New media has also created a revolution in health services. The Internet and social media are being used as reference guides for sensitive health issues by nonprofessionals and physicians alike [2]. The use of social media in the area of health warrants greater scrutiny because of its consequences for public understanding of health issues. Moreover, when used by physicians and medical students, social media has the potential to affect many practical issues such as the differentiation of professional from private life, patient relations, and medical ethics.

Social media is a form of electronic communication intended to create online communities where the users share information, ideas, personal messages, and other content. Social media sites are systems that allow the composition of general or semi-general profiles within a system with defined rules. These sites often show lists of users who are socially connected with one another and allow one to see others’ actions, social connections, and interactions. Social media can be categorized into five groups: common projects (e.g., Wikipedia), blogs or microblogs (e.g., Blogger, Twitter), content communities (e.g., YouTube), social network sites (e.g., Facebook), and virtual games or social worlds (e.g., HumanSim) [2,3].

Social media is also being used extensively in medicine. One-third of all adults have used the Internet in the diagnosis of a medical condition [4]. Furthermore, one-third of individuals with Internet access have looked at blogs, online news groups, and web sites regarding the medical experiences of others, and 6% of these have contributed content through comments, messages, photographs, sound files, and health assessments by professionals or institutes [5]. Individuals with chronic diseases or specific illnesses (e.g., multiple sclerosis or celiac disease) are able to take part in online patient communities in which they can share experiences and treatment options, contact physicians and other patients, and obtain detailed information regarding their diseases. Physicians are also increasingly using social media both professionally and in their daily lives [6,7]. A previous study found that 48% of physicians on Twitter have posted links to their blogs [8]. Comprehensive wikis and webpages, such as AskDrWiki.com where patients can receive information about various diseases and pose questions to specialists, have been created by doctors [2]. Physicians also use social media to exchange information about professional problems and clinical experiences [9,10].

Social media use is common among medical students as they start their professional career; it enables the sharing of information and communication [11]. Thus, the issue of professionalism is pertinent to the use of social media by physicians, and as a result, there have been numerous discussions regarding professionalism in medicine in medical education literature in recent times. Despite the continuing debate over the definition of professionalism, the general aim is to ensure public confidence in the medical profession [12]. For physicians and medical students using social media, ethical sensitivity in their relationships with patients is very important. The matching of patients and physicians via social media and in the public domain does entail some risks, and may lead to speculation and misunderstanding [9]. Both the American and British Medical Associations have put forward recommendations and guidelines for the professional use of social media tools by physicians and medical students to mitigate such problems [13,14].

Social media has presented medical education training strategies in a new dimension. Many educational environments support traditional face-to-face training models via social media seminars, small-group work, and one-on-one mentoring; educators also use blogs for teaching and communication with students [15].

As alluded to above, various issues surround the use of social media in medicine. Some are specific to medicine, such as its use in health services, professional development, communication with patients, and ethical implications. Others concern the use of social media in general, such as ease of use, access, and information exchange. The purpose of this cross-sectional study was to evaluate medical students’ attitudes regarding issues germane to the use of social media in medicine.

## Methods

### Participants

The survey was distributed to all medical students from Afyon Kocatepe University, Faculty of Medicine (Afyonkarahisar, Turkey) who expressed an interest in participating after receiving information about the study. Of the 681 students in the Faculty of Medicine, 70.8% (n = 482) participated in the survey between April and June 2014.

### Measures

The survey consisted of two sections. The first section contained items assessing the frequency of social media use. In this section, medical students indicated whether they used social media for personal or professional purposes, how frequently they used social media sites (never, rarely, several times a month, several times a week, and daily), their academic year, and gender. The second section contained items assessing attitudes toward social media use in medicine. In this section, “the assessment scale of social media usage in medicine” was prepared using existing literature on this topic. Items in this section were in the form of statements to which participants’ responses were measured using a five-point Likert scale (ranging from 1 = “I definitely don’t agree” to 5 = “I definitely agree”).

A preliminary survey using the composed survey form was carried out on 32 physicians. We assessed how long it took to fill out, the understandability of questions, and the relationships among different items (determined using factor analysis). Before being administered to the students, the results of this assessment were used to modify the survey.

Descriptive statistics, a chi-square test, factor analysis, and structural equation modeling were used to analyze the data. A significance level of p < 0.05 was used. Statistical analysis was conducted using the computer programs SPSS 20.0 and Lisrel 8.7.

### Ethical approval

This study was approved by the Board of Directors of Afyon Kocatepe University Medical School. The survey form was distributed in the classroom after the students had been informed. Data was accessible only to the researchers and individual respondents.

## Results

We interviewed 482 students in the Faculty of Medicine at Afyon Kocatepe University: 56.2% students were in their first 3 years (n = 271), 43.8% were in their final 3 years (n = 211), 52.7% were female (n = 254), and 47.3% were male (n = 228). Furthermore, 93.4% of the students used social media (n = 450), and 89.3% used social media for professional purposes (n = 402) (Table 1).

**Table 1:** **Demographic characteristics and social media use by medical students**

Variables	n (%)
Academic year	
First year	110 (22.8)
Second year	70 (14.5)
Third year	91 (18.9)
Fourth year	94 (19.5)
Fifth year	78 (16.2)
Sixth year	39 (8.1)
Gender	
Female	254 (52.7)
Male	228 (47.3)
Social media use	450 (93.4)
Professional social media use	402 (89.3)

The relationship between medical students’ characteristics and social media usage status are provided in Table 2. The overall use of social media was not significantly related to gender (p = 0.434) or academic year (p = 0.549). Similarly, professional social media usage was not significantly related to gender (p = 0.064) or academic year (p = 0.076).

**Table 2:** **Relation of social media and professional social media usage and some variables**

	Using social media	Not using social media			Using social media professionally	Not using social media professionally		
Variables	n (%)	n (%)	*χ* ^ 2 ^	p	n (%)	n (%)	*χ* ^ 2 ^	p
**Gender**								
Female	235 (52.2)	19 (59.4)	0.613	0.434	19 (36.9)	216 (53.7)	3.440	0.064
Male	215 (47.8)	13 (40.6)			29 (60.4)	186 (46.3)		
**Academic year**								
Preclinical (1st, 2nd, 3rd year)	251 (55.8)	20 (62.5)	0.549	0.459	21 (43.8)	230 (57.2)	3.151	0.076
Clinic (4th, 5th, 6th year)	199 (44.2)	12 (37.5)			27 (56.2)	172 (42.8)		

**Figure 1:**
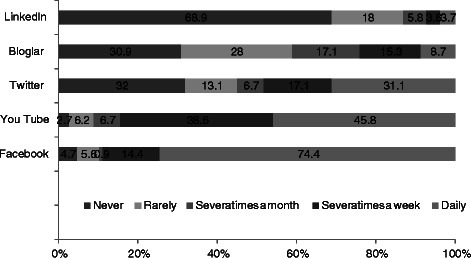
**The distribution of use frequency of social media sites by medical students.**

The distribution of usage frequency of social media sites used by students is provided in Figure 1. We found that 97.3% of students used YouTube, 95.3% used Facebook, 69.1% used blogs, and 68.04% used Twitter. A total of 68.9% of the students reported that they never used LinkedIn, and the majority of students used Facebook daily (74.4%).

### Structural equation model

A structural equation model was created using the responses to items assessing attitudes toward social media. The internal consistency (Cronbach’s alpha) of the scale was α = 0.712. Before proceeding with factor analysis, the conformity of the data was tested (Keiser-Meyer-Olkin coefficient = 0.831, Bartlett’s test of sphericity p < 0.001). Then, the measurement model of the study was determined through confirmatory factor analysis based on the highest probability method. In determining the implicit variables predicting the observed variables based on theoretical assumptions, a varimax conversion was applied and five implicit variables were determined by the calculated basic variables analysis method. Factors that did not meaningfully support these implicit variables and those with factor loadings of less than 0.600 were omitted from the model. The other 15 factors consisted of the observed variables, which were the responses to the specific items (Table 3). Implicit variables were named in accordance with the specifications of these observed variables in the model and consisted of usefulness, popularity, ethics, barriers, and personal innovativeness. The goodness-of-fit values of the model were as follows: root mean square error of approximation (RMSEA) = 0.062, index root mean square residual (SRMR) = 0.043, normed fit index (NFI) = 0.94, non-normed fit index (NNFI) = 0.95, confirmatory fit index (CFI) = 0.96, goodness-of-fit index (GFI) = 0.94, and adjusted GFI (AGFI) = 0.91. Thus, the goodness-of-fit of the model was deemed suitable, and thus the structural equation model was accepted.

**Table 3:** **Factor analysis of assessment scale of social media use in medicine**

		Factor loading				
Factors	Items	1	2	3	4	5
(**1**) Usefulness	Today, the use of social media in medicine is a necessity	0.672				
	Social media is a platform where the recycling of quality information is ensured	0.719				
	Social media is an easy way of knowledge acquisition	0.641				
	The sharing of current information regarding medicine through social media is important for the professional development of physicians	0.781				
	Social media use helps improve the quality of healthcare	0.774				
	Social media contributes to creativity in our profession	0.744				
(**2**) Popularity	Physicians actively using social media are more popular among their colleagues		0.750			
	Popularity in social media enables access to more patient groups		0.778			
(**3**) Ethics	Social media brings along professional or legal risks for physicians			0.788		
	It is hard for patients to differentiate healthy and reliable medical information from incorrect or groundless information			0.832		
(**4**) Barriers	I am too busy to participate in social media				0.717	
	I don’t have time to learn social media usage intended for professional purposes				0.812	
	If I start using it, I am concerned that social media will take a lot of time				0.649	
(**5**) Personal Innovativeness	I actively seek new ways to use social media in my practice					0.811
	I usually find out about new social media applications					0.699

The highest positive correlation in the model was observed between usefulness and personal innovativeness (r = 0.63). This was followed by correlations between usefulness with popularity (r = 0.56) and popularity with personal innovativeness (r = 0.54). The barrier factor was negatively correlated with usefulness (r = −0.34), popularity (r = −0.20), and personal innovativeness (r = −0.270). Ethics had a low but positive correlation with other factors (Table 4).

**Table 4:** **Correlation coefficients among implicit variables based on structural equation modeling**

	1	2	3	4	5
**(1)** Usefulness	1				
**(2)** Popularity	0.56	1			
**(3)** Ethics	0.08	0.17	1		
**(4)** Barriers	–0.34	–0.20	0.03	1	
**(5)** Personal Innovativeness	0.63	0.54	0.18	–0.27	1

## Discussion

This study assessed social media usage in medicine by medical students in Turkey. We found that 93.4% of students used social media and 89.3% used social media for professional purposes. We also found that 95.3% used Facebook; the observed usage rates of Facebook, a social networking site whose use has been investigated in many studies, ranged from 13% to 47% among physicians and from 64% to 96% among students [16].

According to the results of our survey, students perceived social media to have numerous advantages, including inspiring creativity, facilitating professional development, communication with colleagues, knowledge acquisition, and improving the quality of care. An earlier study reported that 24.1% of physicians use social media on a daily basis to search for or explore medical information and 14% contribute to social media on a daily basis [17]. Physicians and medical students commonly use Wikipedia to obtain medical information and specify ease of use as their reason for doing so [18,19]. Many physicians believe that the professional use of social media allows for beneficial information exchange and is useful in caring for patients [18]. Furthermore, many indicated that it was expected that they would be available through social media as their patients were also social media users [20].

Patients also use social media to seek health information, especially patients with serious illnesses, as online resources are primarily for health professionals [21]. Patients also use online sources for the selecting physicians and hospitals [22]. Social media allows physicians to reach a large audience and can act to increase their popularity among colleagues and patients. Furthermore, it can enhance the professional reputation of physicians when used skillfully [23]. In our study, medical students placed importance on the popularity factor, which was positively correlated with usefulness and personal innovativeness.

Our survey results indicated that students who had innovative features were more likely to be social media users. Today’s students, both medical and nonmedical, think and process information fundamentally differently from their predecessors. They represent the first generation to grow up with this new digital technology [24]. However, as shown in the present study, the use of social media by students is also limited by the concern that it will take up too much time [17,19].

In our study, we found no negative effects with respect to ethical values, indicating that students are unaware of ethical issues. The findings of most concern in other studies on online professionalism are incidents of patient privacy violations [25]. In one study, medical school deans reported that medical students had engaged in or posted the following content online: patient confidentiality violations, profanities, discriminatory language, depictions of intoxication, and sexually suggestive material [26]. Medical students may not be aware of the negative effect that posting material online could have on their careers or on medical professionalism in general [26]. Furthermore, such action could result in their peers and other physicians passing judgment on them [27]. While medical students and doctors are entitled to a private personal life, online social media have challenged the concepts of “public” and “private”. Once information is online it is nearly impossible to remove and can quickly spread beyond one’s control [7].

Medical students are expected to develop the same professional ethics as doctors [7]. Therefore, physicians’ associations and medical educators should prepare social media user guidelines for medical students as physicians. However, physicians and students are separated by a generation gap, and thus this is an area of medical education that needs to be specifically targeted [28].

Current medicine practices are being continuously improved as new technologies are developed and implemented. Social media has already transformed the communication sector and is now on its way to transforming healthcare. Social media provides significant opportunities for health professionals, while challenging the traditional core values of medicine (privacy, confidentiality, one-on-one interactions, and formal conduct). To ensure that such improvements continue, it is necessary for physicians to continue to include humanism, honesty, ethics, professionalism, and trust in basic medical values for enhanced and effective patient care [29,30].

### Limitations of the study

This study has several limitations. First, this was a single-institution study. Therefore, it cannot be generalized to all medical students. Second, the use of social media in medicine was evaluated as a whole; therefore, other applications of social media have not been considered here.

## Conclusions

Social media has wrought a revolution in health, as it has in many other fields, and may lead to a future where patients play a greater role in health services. As long as physicians use social media in their practice in an ethical and professional manner, they can benefit both patients and colleagues, enhance their own reputations, and help to lead the revolution in this area. Medical students should also be included in this revolution. If social media is used correctly by medical students, it will contribute to both their education and professionalism. Therefore, it is essential that guidelines for the professional use of social media are produced in the medical field.

## References

[1] Lister M, Dovey J, Giddings S, Grant IH, Kelly K. New Media: A Critical Introduction*.* Second ed. Routledge;2009*.*

[2] Denecke K, Nejdl W (2009). How valuable is medical social media data? Content analysis of the medical web. Inform Sci.

[3] Hamm MP, Chisholm A, Shulhan J, Milne A, Scott SD, Given LM (2013). Social media use among patients and caregivers: a scoping review. BMJ Open.

[4] Fox S, Duggan M. Health Online 2013. Pew Internet & American Life Project. 2013. [http://www.pewinternet.org/Reports/2013/Health-online.aspx]

[5] Fox S. Peer-to-Peer Healthcare. Pew Internet & American Life Project. 2011. [http://pewinternet.org/Reports/2011/P2PHealthcare.aspx]

[6] Lewis P. 86% of Physicians Use Internet to Access Healthcare Information. American Medical News. [http://www.amednews.com/article/20100104/business/301049966/7/]

[7] Mansfield SJ, Morrison SG, Stephens HO, Bonning MA, Wang SH, Withers AH (2011). Social media and the medical profession. Med J Aust.

[8] Chretien KC, Azar J, Kind T (2011). Physicians on Twitter. JAMA.

[9] Hyman JL, Howard JL, Sechrest R (2012). Online professional networks for physicians: risk management. Clin Orthop Relat Res.

[10] Anikeeva O, Bywood P (2013). Social media in primary health care: opportunities to enhance education, communication and collaboration among professionals in rural and remote locations did you know? Practical practice pointers. Aust J Rural Health.

[11] Thompson LA, Dawson K, Ferdig R, Black EW, Boyer J, Coutts J (2008). The intersection of online social networking with medical professionalism. J Gen Intern Med.

[12] MacDonald J, Sohn S, Ellis P (2010). Privacy, professionalism and Facebook: a dilemma for young doctors. Med Educ.

[13] American Medical Association. AMA Policy: Professionalism in the Use of Social Media [http://www.ama-assn.org/ama/pub/physician-resources/medical-ethics/code-medical-ethics/opinion9124.page]

[14] British Medical Association Using Social Media. Practical and Ethical Guidelines for Doctors and Medical Students [http://bma.org.uk/-/media/Files/PDFs/Practical%20advice%20at%20work/Ethics/socialmediaguidance.pdf]

[15] Kind T, Patel PD, Lie D, Chretien KC (2014). Twelve tips for using social media as a medical educator. Med Teach.

[16] Von Muhlen M, Ohno-Machado L (2012). Reviewing social media use by clinicians. J Am Med Inform Assoc.

[17] McGowan BS, Wasko M, Vartabedian BS, Miller RS, Freiherr DD, Abdolrasulnia M (2012). Understanding the Factors That Influence the Adoption and Meaningful Use of Social Media by Physicians to Share Medical Information. J Med Internet Res.

[18] Hughes B, Joshi I, Lemonde H, Wareham J (2009). Junior physician’s use of Web 2.0 for information seeking and medical education: a qualitative study. Int J Med Inform.

[19] Kritz M, Gschwandtner M, Hanbury A, Samwald M (2013). Utilization and perceived problems of online medical resources and search tools among different groups of European physicians. J Med Internet Res.

[20] Glick PL, Yamout SZ (2012). Social media for surgeons: Understand it, embrace it, and leverage it for our profession and our patient. Surgery.

[21] Galarce EM, Ramanadhan S, Viswanath H, Thompson TL, Parrott R, Nussbaum JF (2011). Health information seeking. The Routledge handbook of health communication.

[22] PEW Research Center. Social life of health information [http://www.pewinternet.org/Reports/2011/Social-Life-of-Health-Info.aspx]

[23] Leiker M (2011). When to “friend” a patient: social media tips for health care professionals. WMJ.

[24] Prensky M, Baurlein M (2011). Digital natives, digital immigrant. The digital divide.

[25] Chretien KC, Goldman EF, Beckman L, Kind T (2010). It’s your own risk: medical students’ perspectives on online professionalism. Acad Med.

[26] Chretien KC, Greysen SR, Chretien JP, Kind T (2009). Online posting of unprofessional content by medical students. JAMA.

[27] Rocha PN, de Castro NAA (2014). Opinions of students from a Brazilian medical school regarding online professionalism. J Gen Intern Med.

[28] Osman A, Wardle A, Caesar R (2012). Online professionalism and Facebook–falling through the generation gap. Med Teach.

[29] Chretien KC, Kind T (2013). Social media and clinical care: ethical, professional, and social implications. Circulation.

[30] Gholami-Kordkheili F, Wild V, Strech D (2013). The impact of social media on medical professionalism: a systematic qualitative review of challenges and opportunities. J Med Internet Res.

